# Perinatally administered losartan augments renal ACE2 expression but not cardiac or renal Mas receptor in spontaneously hypertensive rats

**DOI:** 10.1111/jcmm.12573

**Published:** 2015-03-12

**Authors:** Jan Klimas, Michael Olvedy, Katarina Ochodnicka-Mackovicova, Peter Kruzliak, Sona Cacanyiova, Frantisek Kristek, Peter Krenek, Peter Ochodnicky

**Affiliations:** a Department of Pharmacology and Toxicology, Faculty of Pharmacy, Comenius UniversityBratislava, Slovakia; b Department of Cardiovascular Diseases, International Clinical Research Centre, St. Anne′s University Hospital and Masaryk UniversityBrno, Czech Republic; c Institute of Normal and Pathological Physiology, Centre of Excellence for Cardiovascular Research, Slovak Academy of SciencesBratislava, Slovakia

**Keywords:** hypertension, ventricular hypertrophy, losartan, treatment, ACE2/Ang-(1-7)/Mas receptor axis, gene expression

## Abstract

Since the identification of the alternative angiotensin converting enzyme (ACE)2/Ang-(1-7)/Mas receptor axis, renin-angiotensin system (RAS) is a new complex target for a pharmacological intervention. We investigated the expression of RAS components in the heart and kidney during the development of hypertension and its perinatal treatment with losartan in young spontaneously hypertensive rats (SHR). Expressions of RAS genes were studied by the RT-PCR in the left ventricle and kidney of rats: normotensive Wistar, untreated SHR, SHR treated with losartan since perinatal period until week 9 of age (20 mg/kg/day) and SHR treated with losartan only until week 4 of age and discontinued until week 9. In the hypertrophied left ventricle of SHR, cardiac expressions of Ace and Mas were decreased while those of AT1 receptor (Agtr1a) and Ace2 were unchanged. Continuous losartan administration reduced LV weight (0.43 ± 0.02; *P* < 0.05 *versus* SHR) but did not influence altered cardiac RAS expression. Increased blood pressure in SHR (149 ± 2 in SHR *versus* 109 ± 2 mmHg in Wistar; *P* < 0.05) was associated with a lower renal expressions of renin, Agtr1a and Mas and with an increase in ACE2. Continuous losartan administration lowered blood pressure to control levels (105 ± 3 mmHg; *P* < 0.05 *versus* SHR), however, only renal renin and ACE2 were significantly up-regulated (for both *P* < 0.05 *versus* SHR). Conclusively, prevention of hypertension and LV hypertrophy development by losartan was unrelated to cardiac or renal expression of Mas. Increased renal Ace2, and its further increase by losartan suggests the influence of locally generated Ang-(1-7) in organ response to the developing hypertension in SHRs.

## Introduction

Agents modulating renin-angiotensin system (RAS), including AT_1_ receptor blockers (ARB), belong to the most efficacious and widely used class among all antihypertensive drugs because they can target both elevated blood pressure and the progression of organ damage 1. While the efficacy of ARBs in developed hypertension is confirmed, role of the RAS in development and progression of hypertension is not yet fully characterized 2,3. In spontaneously hypertensive rats (SHR), a short-term treatment with ACEIs or ARBs during perinatal period (*i.e*. during intrauterine development and lactation) can provide a long-term protection against cardiovascular morbidity, such as elevated blood pressure and myocardial hypertrophy 4,5; however, mechanisms underlying prevention of spontaneous hypertension and associated organ damage by short-term inhibition of RAS are not fully elucidated. The view of RAS regulation has changed in last two decades because of newly described components of RAS axis consisting of angiotensin converting enzyme 2 (ACE2), angiotensin 1-7 (Ang-(1-7)) and Mas receptor and antagonising the original RAS axis 6,7. Thus, the perception of RAS as a simple cascade leading to the formation of angiotensin II (Ang II) by ACE and exerting its action by AT_1_ and AT_2_ receptors 8 has been replaced by concept of complex regulatory network with several cross-signalling mechanisms. Discovery of local RAS in vascular system, heart and kidneys, which are produced and regulated independently of systemic RAS, is yet another aspect contributing to its complexity 6,7.

Probably one of the most important newly characterized components of RAS is ACE2, isoform of ACE, which, in contrast to ACE, catalyses cleavage of Ang II into Ang-(1-7), a peptide with various cardiovascular effects that are mediated through binding to its receptor Mas 6,7. The balance between the activities of ACE and ACE2 and thus also a balance between Ang II and Ang 1-7 can be crucial in the regulation of local RAS in kidneys, vessels and heart. There are also data showing that ACE2 gene is localized on locus associated with hypertensive phenotype 9; hence, it could play a role in development of spontaneous hypertension.

Across the cardiovascular system, a tight interaction between the RAS and other pathways, including nitric oxide signalling cascade has been observed 10,11. Nitric oxide signalling pathway is relevant in the development of hypertension and consequent hypertensive organ damage 12. Although young SHRs are free of renal injury until late adulthood, the influence of nitric oxide signalling on renal tissue at this age is well described in this model 13. However, while developing cardiac hypertrophy, little is known about nitric oxide signalling in early stages of hypertension development in the SHR.

Spontaneously hypertensive rats are a widely used experimental model to study development of hypertension and consequent functional alterations in cardiovascular system 14,15. While being normotensive until the 4th week after birth, they show a stepwise significant rise of systolic blood pressure (sBP) from the 4th to 9th postnatal week when compared to normotensive controls 15. Interestingly, LV hypertrophy is already present during this period 16. In contrary, the hypertensive renal injury occurs several months later 17 and it can be viewed as a consequence of hypertension in this model. As mentioned above, antihypertensive treatment can effectively prevent hypertension in SHR, as well as consequent pathological alterations of the cardiovascular system, provided that the treatment had begun before development of blood pressure increase 15. Importantly, SHRs are characterized by RAS dysregulation on systemic and also on local level, increased activity of RAS has been reported in heart, kidneys and vessels 18. Accordingly, RAS-modulating agents have significant and consistent antihypertensive and antihypertrophic effects in the SHR 14,15.

Variations in cardiac and renal expression of ACE2/Ang-(1-7)/Mas receptor axis components in the period of period of spontaneous hypertension development are not fully understood. It also remains to be elucidated, whether transient and continuous therapeutic intervention of RAS can modulate potential variations in levels of these components and whether RAS blockade can be responsible for protective effects in cardiovascular system. In our study, we have suggested that losartan, an angiotensin AT_1_ receptor blocker, has an early effect on the components of RAS in the SHR.

## Materials and methods

### Animals and experimental design

Studies were performed on SHR and normotensive Wistar rats (Department of Toxicology and Laboratory Animal breeding, Dobra Voda, Slovak Republic). Rats were kept under standard conditions and received food and water *ad libitum*. For breeding purposes, individual pairs of male and female rats were housed in cages. Once the female rat was believed to be pregnant, her male partner was removed from the cage. The pregnant SHR females were then fed with one of two regimens: (*i*) losartan-enriched chow (losartan 20 mg/kg/day) or (*ii*) regular chow. The losartan dose was adjusted every second to third day according to the feeding habits and bodyweights of the animals. The offspring were weaned not later than at 4th week of age and housed with siblings of the same sex with not more than four animals per cage. Only male rats were further used for purpose of this study. Losartan-treated SHR were further subdivided according to regimen of treatment as follows: (*i*) animals transiently treated perinatally, but losartan was withdrawn after weaning (*i.e*. at 4th week of age; SHR_L-withdrawn_) and receiving standard chow until termination and (*ii*) animals continuously treated perinatally up to 9th week of age, *i.e*. until termination (SHR-L). Losartan dosage was carefully adjusted to the individual feeding habits and increase in bodyweight of the growing rats. None of the losartan-treated offspring demonstrated any malformation and, also the number of pups from losartan-treated females was not different from controls. Similarly, growth of the losartan-treated rats did not differ from their respective controls. Control rats (SHR as well as Wistar) received standard chow during the whole experiment.

All experimental procedures involving the use of experimental animals were approved by the State Veterinary and Food Administration of the Slovak Republic. The investigation conforms to the *Guide for the Care and Use of Laboratory Animals: Eight Edition* (2010) published by the US Committee for the Update of the Guide for the Care and Use of Laboratory Animals; National Research Council, to the EU adopted *Directive 2010/63/EU of the European Parliament and of the Council on the protection of animals used for experimental and other scientific purposes* and *Guide for the Use of Laboratory Animals* (1995) of the Ethics Committee for Experimental Work, Slovak Academy of Sciences.

### Electrocardiography

At the age of 8th week, standard 12-lead electrocardiograms were recorded using needle electrodes placed subcutaneously in rats under tribromoethanol (15 ml/kg, intraperitoneally) anaesthesia. Electrocardiographic recordings were analysed as reported previously 19. Six consecutive beats were evaluated, and the arithmetic means of RR and QT were obtained. Measured QT was corrected to RR duration using Bazett’s equation normalized to resting rat cardiac cycle – QTc = QT/(RR/150)^1/2^ 19.

### Blood pressure measurement and sample collection

From the fourth week of age, arterial sBP, as a measure of hypertension, was measured by the tail-cuff plethysmographic method in pre-trained conscious animals pre-warmed in thermostatic cages 14. Measurements were repeated several times and the mean of six consecutive values after stabilization was taken. Following the last sBP measurement at age of 9th week, animals were sacrificed in carbon dioxide environment and the left ventricles of the heart and kidneys were blotted dry and weighed. The LV weight to bodyweight ratio (LVW/BW) and the kidney weight to bodyweight ratio (Kidney/BW) were calculated. Tissue samples were frozen in liquid nitrogen and stored at −80°C until further processing.

### RNA isolation and quantitative RT-PCR

RNA isolation and quantitative RT-PCRs were performed as previously described 20. Total RNA was isolated from left ventricle and from renal cortices using Tri-Reagent (Ambion, Austin, TX, USA), verified to be intact using agarose gel electrophoresis and reverse-transcribed to cDNA (cDNA RT kit; Applied Biosystems, Foster City, CA, USA). Real-time PCR was performed with SYBR Green detection (Power SYBR Green Master Mix, Applied Biosystems) on ABI Prism 7300 (Applied Biosystems) according to the manufacturer’s instructions. Expression of components of the local renin–angiotensin system was determined using the gene-specific primers given in Table1. β2-Microglobulin (B2m) was used as a reference housekeeping gene. All primers were verified to yield a single PCR product with the correct molecular weight and the absence of signal was confirmed when reverse transcription was omitted.

**Table 1:** Primer sequences for quantitative RT-PCR

Gene	GenBank accession number	Primer sequence (5′–3′)	PCR product length (bp)
Ace	NM_012544.1	Forward: TCCTGCTAGACATGGAGACGA	142
		Reverse: CAGCTCTTCCACACCCAAAG	
Ace2	NM_001012006.1	Forward: CGCTGTCACCAGACAAGAA	129
		Reverse: CGTCCAATCCTGGTTCAAG	
Agtr1a	NM_030985.4	Forward: CACACAACCCTCCCAGAAAG	147
		Reverse: TTGGGGCAGTCATCTTGG	
Mas1	NM_012757.2	Forward: CAGAGCTGGGTTTACCTGGA	132
		Reverse: ATGGCTTTCTCCTCAGCAAA	
Ren1	NM_012642.3	Forward: AGACACAGCCAGCTTTGGAC	112
		Reverse: GAATTCACCCCATTCAGCAC	
B2 m	NM_012512.1	Forward ATGGAGCTCTGAATCATCTGG	105
		Reverse: AGAAGATGGTGTGCTCATTGC	

Ace: angiotensin I converting enzyme; Ace2: angiotensin I converting enzyme 2; Agtr1a: angiotensin II receptor, type 1a; Mas1: MAS1 proto-oncogene, G protein-coupled receptor; Ren1: renin; B2m: beta-2 microglobulin.

### Statistical analysis and calculations

Data are expressed as mean ± SEM. Multiple groups were compared by one-way anova followed by the least significant differences *post hoc* test for multiple comparisons. Differences were considered significant at *P* < 0.05. Relative quantification of mRNA expression in RT-PCR experiments was performed by using the 2^−ΔΔCt^ (Ct – threshold cycle) method 21.

## Results

### Continuous but not transient losartan treatment prevents the development of hypertension and LV hypertrophy

**Figure 1:**
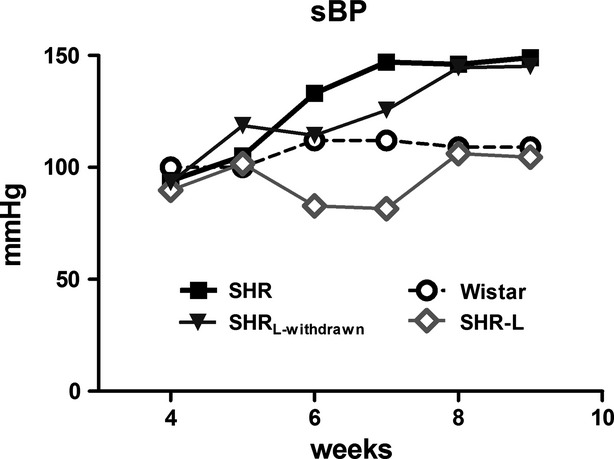
Development of hypertension in SHR and the influence of losartan administration. Means are presented. SHR_L-withdrawn_, transient treatment with losartan until 4th week of age; SHR-L, continuous treatment with losartan.

In spite of similar sBP values in SHR as compared to Wistar at the weaning period, hypertensive rats displayed a rapid rise of blood pressure during the follow-up period (see Fig.1). Losartan administration prevented this development only when given continuously until the termination of study. Although a tendency to attenuate at the beginning, the transient administration of losartan exclusively during perinatal period was unable to significantly affect sBP during follow-up.

In parallel to hypertension, SHRs developed LV hypertrophy, measured as increased LVW and LVW/BW, when compared to Wistars (see Table2). Other clinical features remained unchanged. Also, we found identical characteristics of SHR_L-withdrawn_ when compared to control SHR.

**Table 2:** Basic characteristics of experimental groups at the termination of study

	Wistar	SHR	SHR_L-withdrawn_	SHR-L
BW (g)	218 ± 9	206 ± 4	199 ± 7	196 ± 6
LVW (g)	0.50 ± 0.03	0.63 ± 0.02*	0.60 ± 0.04*	0.43 ± 0.02#
LVW/BW (mg/g)	2.3 ± 0.1	3.1 ± 0.1*	2.9 ± 0.1*	2.2 ± 0.1#
Kidney	0.81 ± 0.04	0.73 ± 0.03	0.74 ± 0.04	0.66 ± 0.02
Kidney/BW (mg/g)	3.7 ± 0.2	3.5 ± 0.1	3.6 ± 0.1	3.4 ± 0.1
sBP (mmHg)	109 ± 2	149 ± 2*	145 ± 2*	105 ± 3#
QTc (msec.)	81 ± 2	80 ± 2	82 ± 2	79 ± 3

Mean ± SEM (*n* = 8–10 per group

* *P* < 0.05 *versus* CON

# *P* < 0.05 *versus* SHR).

BW: bodyweight; LVW: left ventricular weight; sBP: systolic blood pressure at the 9th week of age; QTc: corrected QT interval.

### mRNA expression of LV RAS components

**Figure 2:**
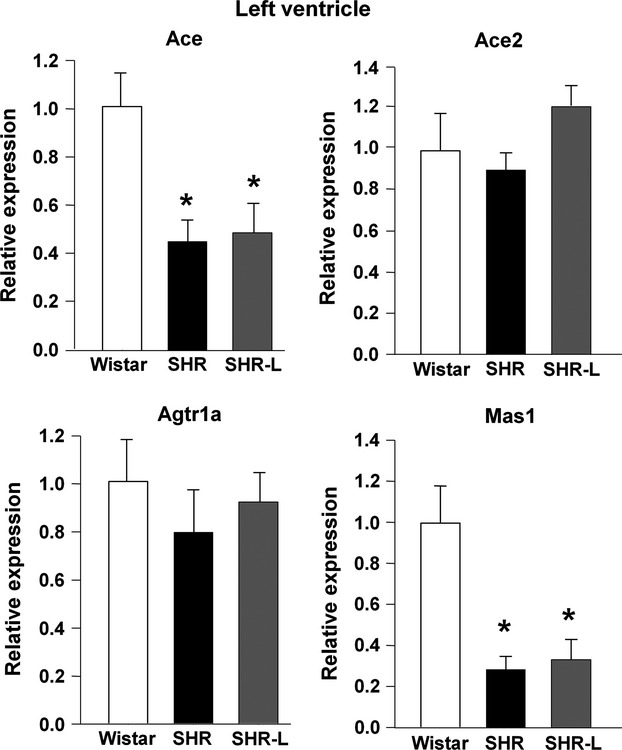
mRNA expression of components of the local renin–angiotensin system in the left ventricle. Ace: angiotensin I converting enzyme; Ace2: angiotensin I converting enzyme 2; Agtr1a: angiotensin II receptor, type 1a; Mas1: MAS1 proto-oncogene, G protein-coupled receptor. Mean ± SEM (*n* = 7?9 per group; *P < 0.05 *versus* CON).

LV hypertrophy in SHR was characterized by a decreased mRNA expression of Ace and Mas receptor genes while no changes were detected in mRNA expressions of Ace2 and of AT_1_ receptor genes (see Fig.2). Neither continuous (see Fig.2) nor transient (data not shown) losartan administration had any influence on these changes.

### Protein expression of nitric oxide signalling components in left ventricle

**Figure 3:**
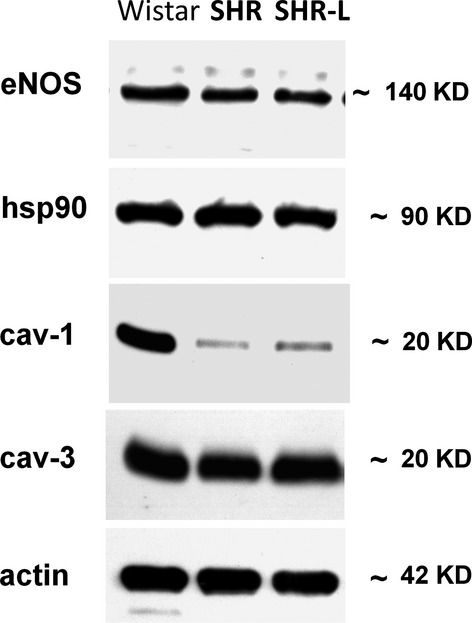
Identification of LV expression of nitric oxide synthases and their allosteric modulators caveolin-1, caveolin-3, and hsp90 at protein level. Actin was used as a loading control. eNOS and iNOS: endothelial and inducible nitric oxide synthase; cav-1 and cav-3: caveolin 1 and 3; hsp90, heat shock protein 90.

We observed a lower expression of cardiac eNOS and cav-1 protein in the left ventricle (see Fig.3 and Table3). Other analysed proteins were unchanged or not detectable (nNOS). Neither continuous (see Fig.3 and Table3) nor transient (data not shown) losartan administration had any influence on the found decrease in eNOS and cav-1.

**Table 3:** Cardiac protein expression of nitric oxide-signalling pathway

	Wistar	SHR	SHR-L
eNOS	100 ± 6	78 ± 3*	60 ± 8*
iNOS	100 ± 16	61 ± 14	51 ± 13
nNOS	ND	ND	ND
cav-1	100 ± 6	27 ± 10*	16 ± 7*
cav-3	100 ± 6	84 ± 5	91 ± 6
hsp90	100 ± 8	104 ± 14	98 ± 7
Actin	100 ± 3	92 ± 6	97 ± 5

Mean ± SEM (*n* = 6–8 per group

* *P* < 0.05 *versus* CON).

eNOS: endothelial nitric oxide synthase; iNOS: inducible nitric oxide synthase; nNOS: neuronal nitric oxide synthase; cav-1 and cav-3: caveolin-1 and caveolin-3; hsp90: heat shock protein 90; ND: not detectable.

### mRNA expression of renal RAS components

**Figure 4:**
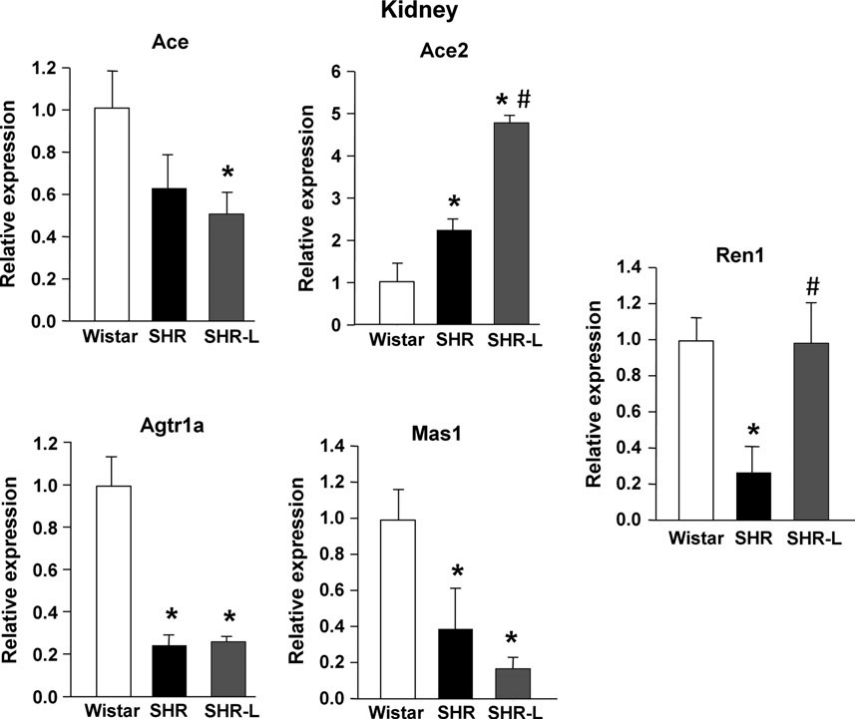
mRNA expression of components of the local renin–angiotensin system components in the kidney. Ace: angiotensin I converting enzyme; Ace2: angiotensin I converting enzyme 2; Agtr1a: angiotensin II receptor, type 1a; Mas1: MAS1 proto-oncogene, G protein-coupled receptor; Ren1: renin. Mean ± SEM (*n* = 7?9 per group; *P < 0.05 versus CON, #P < 0.05 *versus* CON, ^#^*P* < 0.05 *versus* SHR).

As kidneys are substantial regulators of blood pressure, we analysed local RAS components also in renal tissue (see Fig.4). Similar to LV mRNA expressions, we found lower levels of ACE and MAS receptor mRNA in the SHR. However, in contrast to the left ventricle, we observed significantly decreased mRNA level of AT_1_ receptor but a significant increase in ACE2.

When treated until termination of study, losartan significantly potentiated the renal up-regulation of ACE2 and normalized the reduced expression of renin in SHR (Fig.4). Withdrawal of losartan resulted in identical results as observed in non-treated SHR (data not shown).

## Discussion

The main finding of this study is that continuous (perinatal and postnatal) administration of losartan suppressed the development of hypertension and LV hypertrophy but this was not associated with changes in cardiac or renal expression of Mas receptor in SHRs. However, losartan further accelerated exclusively the already increased renal ACE2 expression suggesting influence of Ang-(1-7) in kidneys but not in left ventricles of maturating SHRs. When discontinued after weaning, the effect of losartan administration was not persistent.

As described earlier 2,3, therapy during foetal development can interact with foetal programming for long-term blood pressure, where RAS plays a prominent role by ensuring correct development of mechanisms responsible for long-term blood pressure regulation. Additionally, some authors 4 suggested that even a temporary intervention into RAS with ACEIs or ARBs during the period of developing spontaneous hypertension can, on a long-term basis, prevent the further progression of hypertension and associated organ damage. On the other hand, also detrimental long-term consequences of perinatal suppression of RAS have been described. As shown in rats 22, perinatal blockade of AT_1_ receptors by losartan can result in reduced renal function and increased blood pressure in adulthood. Although there is lack of experience with long-term consequences of use of RAS-modulating drugs in human newborns, their use can trigger renal failure in sick hypertensive premature infants 23 and it is contraindicated during pregnancy because of teratogenicity likely caused by foetal hypotension and renal failure 24. In our study, losartan failed to affect the rapid disease progression following treatment interruption what supports the view that, although RAS may play an important role in the development of hypertensive disease, its transient modulation by losartan exclusively during perinatal period fades after withdrawal 2. However, we still cannot exclude the long-term consequences of perinatal modulation of RAS in adulthood. Unfortunately, the concept of short-term intervention into RAS leading to a long-term antihypertensive protection still remains controversial and requires further research.

We observed changes in mRNA expression in both ACE/Ang II/AT_1_ receptor axis as well as in ACE2/Ang-(1-7)/Mas receptor axis in hypertrophied left ventricles from SHRs. In detail, Ace gene was downregulated while Ace2 gene remained stable. However, responsible receptors did not reflect these changes as AT_1_ receptor remained stable and Mas expression decreased. In general, Ang-(1-7) has been presented as a functional antagonist of Ang II actions in the heart, preventing Ang II-induced cardiac remodelling 25. While Ang II exerts its cardiac action mainly through AT_1_ receptors, actions of ANG-(1-7) are mediated by Mas receptors 7. These results suggest that both local ACE/Ang II/AT_1_ receptor axis as well as local ACE2/Ang-(1-7)/Mas receptor axis are rather silent in cardiac tissue during the development of hypertension and hypertrophy. This is consistent with the findings of others, which reported a rather decreased 26,27 cardiac ACE2 expression and similarly a decreased cardiac MAS expression in hypertensive rats 26. However, other experimental models of developing hypertension, *e.g*. by an infusion of Ang II, exhibited enhanced of ACE2 levels 28 suggesting the importance of Ang II overproduction for Ang-(1-7) arm activation. Our data suggest that rather the markedly suppressed Mas expression as the over-activation of cardiac ACE/Ang II/AT_1_ receptor axis is related to cardiac hypertrophy in early stage of hypertension development in young SHRs.

Apart from their basic function of hindering Ang II binding to AT_1_ receptor, ARBs have been reported to affect also the ACE2/Ang-(1-7)/Mas receptor axis. Namely, increased expression and/or activity of ACE2 was found 25,27, suggesting beneficial influence of ACE2/Ang-(1-7)/Mas receptor axis in cardiac tissue. The explanation of changes in ACE2/Ang-(1-7)/Mas receptor axis is in the increased Ang II concentration following AT_1_ receptor blockade which, in turn, is available as a substrate for ACE2-mediated degradation 29. However, in our study, losartan administration failed to affect cardiac expression of any of RAS components although significantly prevents development of LV hypertrophy, concomitantly to its antihypertensive action. In summary, our results indicate that local Ang II production and/or action is likely not contributing to development of cardiac hypertrophy in early stages of hypertension development and, instead, the reduction in afterload following losartan appears to be the pivotal factor.

The tight interplay between RAS and nitric oxide signalling pathway across cardiovascular system is well known. Importantly, cardiac hypertrophy induced by RAS activation has been demonstrated to be counter-regulated by nitric oxide 10,30. In particular, AngII has been shown to modulate release of nitric oxide through AT_1_ receptors 11,30,31 and, *vice versa*, nitric oxide has been shown to decrease Ang II production and action 30–32. In our young SHRs, changes in cardiac mRNA expression of RAS components (ACE and Mas) were accompanied by a reduction in two crucial components of nitric oxide pathway – eNOS and its negative modulator cav-1. This is in accordance with findings in adult SHRs 12. The coordinated expressional changes in eNOS and its allosteric regulator cav-1 may be viewed as a compensatory mechanism to maintain the production of bioactive nitric oxide in the face of developing LV hypertrophy in the setting of arterial hypertension. Although the reduction in eNOS was moderate, it remains likely that the dramatic decrease by 80% in cav-1 was sufficient to enhance the ability of the residual eNOS to produce nitric oxide in the intact tissue *in vivo*. In fact, already a 25% reduction in caveolin-1 is sufficient to increase basal and agonist-stimulated eNOS activity in intact endothelial cells 12. Thus, although eNOS was reduced, it is conceivable that the decrease in caveolin-1 may lead to hyperactivation of eNOS and elevations of nitric oxide production in the intact heart. In turn, nitric oxide suppresses the synthesis of ACE 33. In this context, one could speculate that the downregulation of Ace gene might be related to an overproduction of nitric oxide in young SHRs.

Also the ACE2/Ang-(1-7)/Mas pathway interacts with nitric oxide pathway and many actions elicited by ANG-(1-7) are mediated by nitric oxide. Costa *et al*. showed an association between ANG-(1-7)-induced decrease in arterial pressure and increase in cardiac NOS (eNOS and nNOS) expression and activity in adult SHR 34. Also Dias-Peixoto *et al*. identified eNOS as a downstream mediator of Ang-(1-7) signalling pathway in cardiomyocytes 35. On the other hand, neither Ang-(1-7) transgenic rats 36 nor Mas knockout mice 35 exhibited any changes in eNOS levels in ventricular myocytes. Thus, the downregulation of Mas in our study is likely being more an epiphenomenon than being in a direct relationship with the decreased expression of eNOS.

Even when administered continuously, losartan failed to affect protein expression of nitric oxide pathway components in hypertrophied ventricles. This is in contrast to other experimental studies, where ARBs (including losartan) were found to increase eNOS expression in cardiac tissue 29. Similarly, when Ang II decreased caveolin1 protein levels 37, consequently, Ang II inhibition should increase it. However, AT_1_ receptor blockade reversed cardiac abnormalities also in Cav-1 knockout mice 38. Taken together, our study suggests that AT_1_ receptor blockade reversed the developing cardiac hypertrophy independently from cardiac nitric oxide pathway during early-phase of hypertension.

Interestingly, in spite of developing arterial hypertension, renal expressions of key renal RAS components leading to an overproduction of Ang II (ACE) and responsible for its harming effect (AT_1_ receptors) has been suppressed in our SHRs. Suppressed expression of AT_1_ has been already observed also by others 39 and this was dependent on phase of hypertension progression. Likely, reduced AT_1_ receptor expression is a compensatory mechanism in a reaction to Ang II production. However, the downregulation of renal ACE is rather controversial. Regulated independently from the circulating RAS, intrarenal formation of ANG II modulates several renal functions. Additionally, kidneys might possess an antihypertensive action that could buffer the pressor actions mediated by the renin dependent formation of ANG II in the circulation 6. Thus, the downregulation of ACE might be explained as compensatory downregulation because of increased ANGII activity in the kidneys.

Renin in the cardiovascular system is primarily kidney-derived as a response to systemic blood pressure and blood volume and its expression can dynamically change during developing hypertension 40. In our young SHRs, we have observed a decrease in its renal expression what was consistent with findings from others 41. One might expect that an increase in the circulating and tissue ANG II suppresses renin release from juxtaglomerular cells and therefore also the production of ANG II by a negative feedback mechanism in the kidney. However, there is evidence that a positive feed-forward loop exists in the kidney under excessive ANG II action, including augmentation of renin 42 and ACE 43. Whether the downregulation of renin in kidneys in young SHRs is a result of excessive or diminished ANGII action must be cleared in future studies. The downregulation of ACE and AT_1_ receptor rather support the view of less locally produced AngII perhaps as a consequence to systemic AngII overload.

In contrast to ACE/Ang II/AT_1_ receptor axis, the complex role of ACE2/Ang-(1-7)/Mas axis in the regulation of renal hemodynamics and in development of kidney damage is still controversial. In isolated blood vessels including the renal afferent arterioles, Ang(1-7) induces a robust vasodilation *via* Mas receptor 44 suggesting its beneficial influence in the renal tissue. Also, the majority of studies described ACE2/Ang-(1-7)/Mas pathway to serve protection 6,7 even independently of blood pressure 45. Nevertheless, also the opposite has been suggested 46,47. The controversy inside this pathway is apparent also in our study. In kidneys of young SHRs, contradictory to similarly suppressed Ang II pathway components, we found differently affected components of Ang-(1-7) axis – ACE2 expression was increased but that of Mas receptor was suppressed. The reasons for this discrepancy are not yet clarified but may be dependent on ‘bimodal’ action of Ang-(1-7) in kidneys – it inhibits Ang II-induced phosphorylation of MAP kinases *via* Mas receptor and, concurrently, stimulates phosphorylation of specific MAP kinases (also *via* Mas receptor), what is similar to the effects of Ang II 47,48.

As ACE2 critically participates on the formation of Ang-(1-7) from Ang II, one might speculate that increased ACE2 in SHRs is a consequence of higher levels of Ang II in kidneys. However, until now, ACE2 levels were consistently reported to be downregulated in kidneys of hypertensive rats 26,27,45. This suggests rather a negative regulatory role of ACE2 in blood pressure control. The increase in renal ACE2 responsible for Ang-(1-7) in our SHRs may suggests that in developing hypertension the ACE2/Ang-(1-7)/Mas receptor axis could compensate increased systemic blood pressure activation. However, in light of renal downregulation of Ang II axis components, we could speculate about compensatory ACE2 up-regulation to decreased local ACE synthesis in kidneys in early hypertensive rats. Also, high levels of ACE2 may be associated with a local increase in the Ang II breakdown system compared to Ang II generation, which may be important for counterbalancing the high systemic arterial pressure.

While some authors proposed a detrimental effect of Mas deficiency because of the inability of Ang-(1-7) to mediate its beneficial effects 49, others described benefits of Mas downregulation in kidney 46. It is of note, Mas receptor expression was downregulated in kidneys of our SHRs, which could suggest a shift of balance within local RAS in favour of Ang II. However, from all components studied, only reduced expression of Mas receptor is suggesting increased Ang II activity, while the modifications in expression of ACE, ACE2 and AT_1_ receptor would lead to suppression of Ang II production or activity.

Interestingly, losartan further significantly potentiated the increase in ACE2, but failed to influence the ACE and Mas downregulation. In addition, losartan normalized renin expression. While up-regulation of renin by losartan might be a compensatory response to systemic blood pressure lowering effect, since renin is expected to be the only RAS component produced mostly on systemic level rather than locally 18, the further augmentation of Ace2 is rather a consequence of local regulation. Indeed, increase in renal ACE2 has been consistently described in response to blockade of AT_1_ receptors and has been related to beneficial influence of ACE2 in kidneys 50.

The rather beneficial effects of ACE2/Ang-(1-7)/Mas axis documented in experimental studies very seriously favour this axis as a promising alternative therapeutic target in hypertension-related target organ damage 51. As hypertension is closely linked to atherogenesis, it is conceivable that ACE2/Ang-(1-7)/Mas axis may significantly modulate also this pathology. Indeed, Ang(1-7) was shown to inhibit atherosclerotic development in apolipoprotein E knockout mice (ApoE^−/−^) probably by complex interactions between Mas receptors and AT_2_ receptors resulting into elevated nitric oxide bioavailability 52,53. Already existing Mas agonists were shown not only to induce vasodilatation, to reduce blood pressure and to suppress kidney as well as cardiac injury in different experimental models (as reviewed by 54) but also to inhibit atherogenesis in ApoE^−/−^ mice 55. This finding might predict their future indications, however, further research in humans is now requested to confirm this concept.

This study has shown that components of RAS are differently expressed in the heart and kidney in settings of developing hypertension. Moreover, losartan affected renal but not cardiac expression of ACE2. It is known that ACE2 expression in the hypertensive heart and kidney is not related to circulating Ang II levels 28. Also our study underlines the different local RAS response to hypertension development.

## Conclusion

Conclusively, although losartan administration prevented the rise of blood pressure as well as the development of LV hypertrophy, this action was not related to changes in cardiac or renal expression of Mas receptor. On the other hand, the demonstration of increased renal Ace2 gene expression, and potential of its further acceleration following AT_1_ receptor blockade already at the early stages of development of hypertension suggests that locally generated Ang-(1-7) may contribute to resistance of kidneys to develop organ damage in SHRs. In other words, interactions inside RAS may play significant role in long-term resistance of kidneys, but not of left ventricles, against chronic hypertension in SHRs. It is of note, that the action of perinatally administered losartan did not persist after treatment discontinuation. Though there might be several limitations preventing us from drawing a clear conclusion, namely missing detection of tissue concentrations for concerned peptides or alternative pathways for Ang II production, our results showing prominent potential for inducible (*e.g*. by AT_1_ receptor blockade) augmentation of Ang-(1-7) generation may be a possible mechanism through which ACE2 may exert renal but not cardiac protection in SHR.
